# Surgical Technique: Viscodissection for Managing Funnel Retinal Detachments

**DOI:** 10.3390/jcm14134394

**Published:** 2025-06-20

**Authors:** David Oliver-Gutierrez, Claudia García-Arumí, Daniel Gómez Plaza, José García-Arumí

**Affiliations:** 1 Department of Ophthalmology, Hospital Universitari Vall d’Hebron, 08035 Barcelona, Spain; claudia.garciaarumi@vallhebron.cat (C.G.-A.); jgarcia.arumi@gmail.com (J.G.-A.); 2 Department of Ophthalmology, Instituto de Microcirugía Ocular (IMO), Miranza, 08035 Barcelona, Spain; daniel.gpl10@gmail.com

**Keywords:** viscodissection, retinal detachment, funnel-shaped retinal detachment, vitreoretinal surgery, proliferative vitreoretinopathy (PVR)

## Abstract

**Purpose**: To evaluate and describe the efficacy and safety of viscodissection in managing complex funnel-shaped retinal detachments, minimizing trauma and facilitating safer perfluorocarbon liquid (PFCL) application. **Methods**: A retrospective case series of five patients with funnel-shaped retinal detachments: three due to perforating trauma and two from recurrent detachments. Initial visual acuities ranged from light perception to hand motion. Viscodissection was used to separate adhered retinal tissues in the funnel-shaped retinal detachment in a controlled, minimally traumatic manner, allowing funnel opening and PFCL application. Data collected included demographics, visual acuities, surgical details, and complications. **Results**: Viscodissection enabled successful funnel opening and PFCL use in all cases, with one instance of subretinal migration of PFCL. No retinal detachment recurrences occurred, but three patients required reoperation for new premacular proliferative vitreoretinopathy (PVR). Postoperative visual acuities improved in four patients (up to 20/100), while one remained at hand motion. **Conclusions**: Viscodissection is a promising technique for complex funnel-shaped retinal detachments, allowing non-traumatic tissue separation and improving visualization and safety during PFCL application. This approach may enhance surgical outcomes and reduce complications.

## 1. Introduction

Retinal detachment associated with proliferative vitreoretinopathy (PVR) remains one of the most challenging vitreoretinal conditions, often leading to surgical failure due to tractional forces and fibrotic membrane formation [1]. Among its most complex presentations, funnel-shaped retinal detachments represent an advanced stage where circumferential contraction and fibrocellular proliferation create a rigid, non re-attachable retinal architecture [2].

Conventional techniques, including vitrectomy, membrane peeling, and PFCL use, may be insufficient due to strong adhesions between the retina and epiretinal or subretinal membranes, with the added risk of subretinal PFCL migration. Subretinal migration occurs when PFCL passes through retinal breaks or areas of retinal thinning, particularly in the presence of significant traction, allowing it to seep into the subretinal space. Therefore it is standard practice to fist locate the optic nerve head and drip PFCL over it.

Viscodissection offers a controlled method for opening the funnel and locating the optic nerve head. It is a minimally traumatic alternative, especially in cases where mechanical dissection alone poses a risk of further retinal damage. Injecting a viscoelastic substance into the funnel facilitates its opening, allowing visualization of the optic nerve head and enabling safer PFCL application and PVR peeling.

This article describes the surgical technique of viscodissection and its role in complex funnel retinal detachments, where standard surgery alone may be inadequate. By enhancing intraoperative visualization, minimizing tissue trauma and allowing safer PFCL use, viscodissection improves surgical outcomes.

## 2. Methods

Medical records of the past 5 years were accessed for research purposes in January 2025. During data collection, the authors had access to identifiable patient information; however, all data were anonymized prior to database construction and analysis. We retrospectively reviewed the clinical records of patients who underwent surgery for funnel retinal detachment using viscodissection. Collected data included demographics, baseline and final ophthalmic findings, surgical details, and complications during follow-up.

This study complies with the principles of the Declaration of Helsinki, and all patients provided written informed consent for both the surgical procedure and the use of de-identified data for research and publication.

### Surgery Technique: Viscodissection for Funnel Retinal Detachment (Figure 1)

After maximal vitreous removal, if a funnel retinal detachment is present and PFCL poses a risk of subretinal migration, while traction with metallic instruments could cause further retinal damage, viscodissection becomes a safer alternative.

Viscodissection is performed using a straight silicone-tip cannula connected to a tubing system for enhanced manual control and a syringe filled with sodium hyaluronate 10 mg/mL (Healon^®^). The viscoelastic is injected slowly into the funnel center, progressively separating the retinal walls.

Once the funnel is sufficiently open and traction relieved, the risk of PFCL migration is significantly reduced. PFCL is then injected near the optic disc, beneath the viscoelastic, which is displaced anteriorly. This stabilizes the retina and facilitates PVR peeling and proper re-attachment.

**Figure 1:**
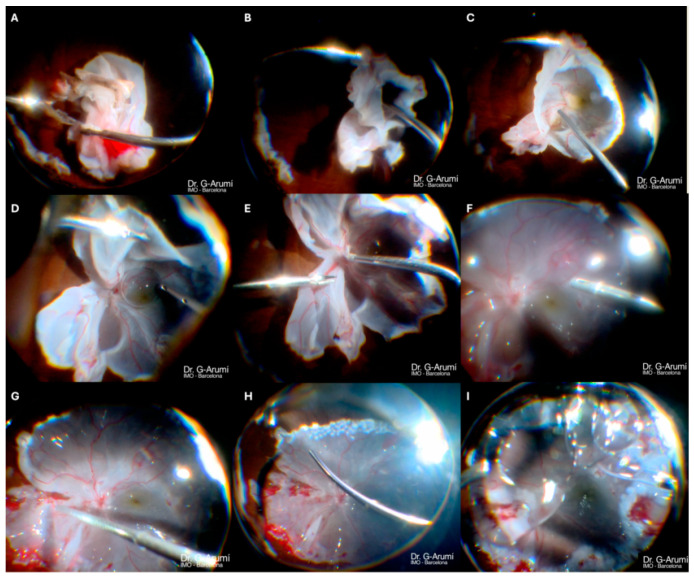
Step-by-step description of funnel-shaped retinal detachment surgery using viscodissection. (**A**) Bimanual peeling of subretinal PVR. (**B**) A silicone-tip cannula connected to a viscoelastic syringe is inserted into the funnel. (**C**) Viscodissection facilitates the opening of the funnel and allows for proper visualization of its inner part. (**D**) PFCL is safely placed at the interface between the retina and viscoelastic, in front of the papilla. (**E**) Bimanual peeling of preretinal PVR. (**F**) Additional injection of PFCL. (**G**) The retina is carefully extended and positioned with the help of a silicone-tip cannula. (**H**) 360-degree endophotocoagulation (EFCG) of the retinotomy. (**I**) Injection of silicone oil (These images are taken from the Video S2 of case 5 described later in this paper).

## 3. Results

We report five cases in which Dr. J.G-A. used viscodissection to manage funnel retinal detachments. Three resulted from perforating trauma, and two were recurrences of previously treated detachments. Initial visual acuity was light perception (LP) in one patient and hand motion (HM) in four. Viscodissection effectively opened the funnel and enabled safe PFCL use in all cases, with only one instance of subretinal migration. No retinal detachment recurrences were observed; however, three patients required reoperation due to new macular PVR. Postoperatively, four patients improved, reaching up to 20/100, while one remained at HM.

### 3.1. Case 1

A 15-year-old female sustained corneal and scleral perforations from direct trauma, requiring emergency suturing. Her initial visual acuity was LP. Two months later, once stable, she underwent pars plana vitrectomy (PPV) with viscodissection to separate the partially incarcerated retina and open the funnel. PFCL was applied to stabilize the retina, followed by preretinal PVR peeling, 360-degree endophotocoagulation (EPCG), and 5000 cSt silicone oil (SO) tamponade. Prolene retention sutures were placed due to aphakia and iris loss.

Six months postoperatively, visual acuity remained HM, with a stable retina, silicone oil, and retention sutures.

### 3.2. Case 2: Figure 2

A 71-year-old male with a history of vitrectomy for retinal detachment four months prior, where SO was used as tamponade, presented with a recurrent detachment. The entire retina was detached, forming a funnel in the lower hemisphere, and his visual acuity had declined to hand motion (HM).

During surgery, SO was removed, and viscodissection was performed to open the funnel. PFCL was then introduced, allowing bimanual PVR dissection and subsequent 360-EPCG. A 5000 cSt SO tamponade was reapplied.

Two months later, a second vitrectomy was performed to remove premacular PVR and subretinal PFCL while maintaining the existing SO tamponade. Subretinal PFCL was aspirated thought a retinotomy. One week postoperatively, the patient’s visual acuity improved to 20/400.

**Figure 2:**
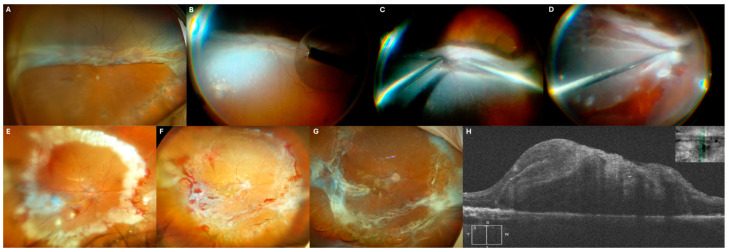
(**A**) Initial presentation at our center with complete retinal detachment and overfolded inferior retina. (**B**) Intraoperative image, showing silicone oil aspiration. (**C**) Intraoperative image, viscodisection to open the overfolded inferior portion of the retina. (**D**) Intraoperative image, inferior retinotomy. (**E**) Retinography 5 days after surgery. (**F**) Progression showing premacular PVR. (**G**) After second surgery for premacular PVR peeling. (**H**) Final OCT indicating absence of subretinal fluid.

### 3.3. Case 3: Figure 3 and Video S1

A 43-year-old male with a history of cataract surgery (two years prior) and vitrectomy for retinal detachment (two months prior) presented with visual acuity of HM. Examination revealed a funnel-shaped retinal detachment. During surgery, a 360-degree scleral buckle was placed, followed by PPV. Viscodissection was used to open the retinal tunnel, enabling bimanual epiretinal membrane dissection and 360-degree EPCG. A 5000 cSt SO tamponade was applied for retinal stabilization.

Four months later, a follow-up surgery was required to remove SO and address newly developed PVR. Two months postoperatively, the retina remained attached, and visual acuity improved to 20/100.

**Figure 3:**
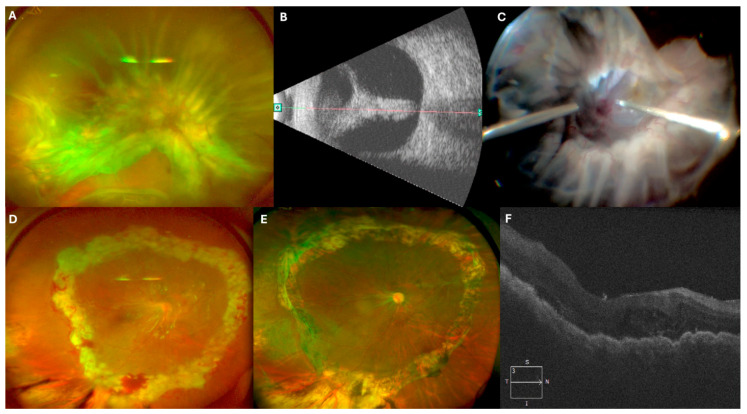
(**A**) Initial presentation with a recurrent funnel-shaped retinal detachment. (**B**) Echography showing the funnel-shaped detachment. (**C**) Intraoperative image displaying viscodissection that allows visualization of the funnel’s inner part, enabling safe use of PFCL. (**D**) Retinography one-week post-surgery. (**E**) Retinography four months post-surgery. (**F**) OCT scan showing the attached retina 4 months post-surgery (Video S1).

### 3.4. Case 4: Figure 4

A 22-year-old male with a history of ocular penetrating trauma presented two months post-injury with visual acuity of HM. Ultrasound revealed a total retinal detachment with a funnel-shaped configuration.

He underwent PPV, where viscodissection was used to expand the funnel, facilitating PFCL application and bimanual PVR dissection. After retinal reattachment and 360-degree EPCG, a 5000 cSt SO tamponade was applied.

Two months later, SO and residual PVR were removed. One year postoperatively, the retina remains attached, and visual acuity has improved to 20/200.

**Figure 4:**
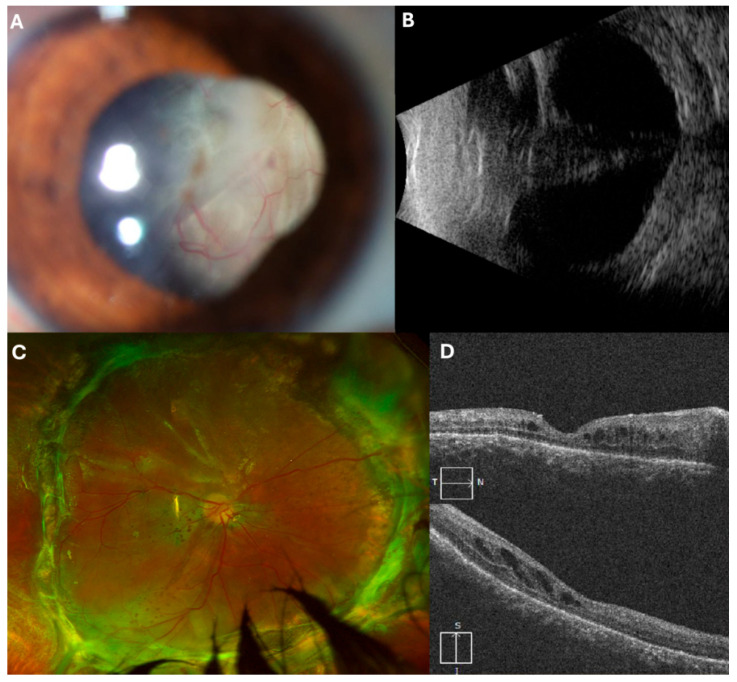
(**A**) Initial presentation with retinal detachment visible in the anterior chamber. (**B**) Funnel-shaped retinal detachment observed on echography. (**C**) Two months post-second surgery. (**D**) OCT scan displaying the attached retina.

### 3.5. Case 5: Figure 1, Figure 5 and Video S2

A 13-year-old male with a history of ocular trauma underwent primary repair, including vitrectomy, at another center. He presented 15 days later with visual acuity of HM, hematocornea, and ultrasound findings of total funnel-shaped retinal detachment. Surgical management included corneal trephination, placement of a temporary Eckardt keratoprosthesis, and viscodissection to release retina-iris adhesions and open the funnel. This facilitated PFCL application and bimanual PVR dissection. Laser EPGC was performed at the retinotomy edges, followed by 5000 cSt SO injection and repositioning of the corneal button.

One month postoperatively, optical coherence tomography confirmed retinal attachment; however, visual acuity remained at HM.

**Figure 5:**
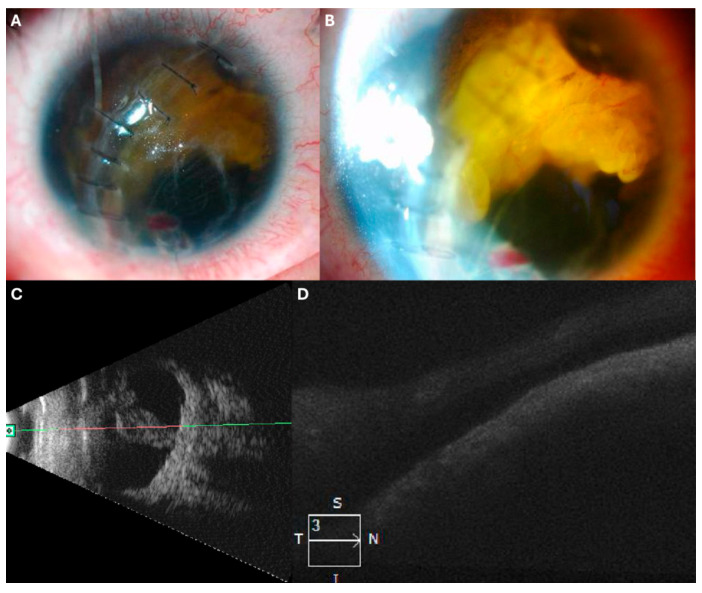
(**A**,**B**): Initial presentation displaying retinal detachment in the anterior chamber during slit lamp examination. (**C**) Funnel-shaped retinal detachment evident on echography. (**D**) OCT scan one month postoperatively showing the retina attached. Figure 1 and Video S2 are also part of this case.

## 4. Discussion

PVR remains a major challenge in retinal detachment surgery, with multiple risk factors, including retinal breaks, choroidal detachment, vitreous hemorrhage, preoperative retinal folds, inflammatory conditions, previous retinopexy, and genetic predisposition [3].

PVR severity varies widely. If the macula remains attached, early surgery is recommended, whereas if detached, some studies suggest delaying intervention until the proliferative phase stabilizes. However, postponing surgery beyond 28 days may increase the risk of PVR progression and poor functional outcomes [4,5].

Advances in wide-field visualization, high-speed vitrectomy probes, and intraoperative staining have significantly improved PVR surgery outcomes [5,6]. Despite this, removing residual vitreous and epiretinal membranes remains challenging, especially in cases with strong vitreoretinal adhesions. PFCL stabilizes the retina, but improper use risks subretinal migration. Triamcinolone acetonide and trypan blue enhance membrane visualization, enabling safer dissection [7].

Viscodissection has been described for separating PVR membranes from the retina, particularly in diabetic patients. This technique involves injecting viscoelastic substances between the retina and epiretinal or subretinal membranes, facilitating separation while minimizing the risk of iatrogenic damage, such as retinal tears or hemorrhages [8,9,10].

Funnel retinal detachments pose significant challenges due to circumferential and anteroposterior contraction, dense fibrotic membranes, and severe retinal stiffness [2]. Conventional approaches, including vitrectomy, membrane peeling, and PFCL-assisted reattachment, may be insufficient due to extreme membrane adherence [5]. In such cases, viscoelastic agents have been employed to push the funnel-shaped retinal detachment posteriorly out of the anterior chamber [11] allowing for easier and safer trocar placement. However, viscodissection of the funnel itself has not been previously described.

Viscodissection represents a key advancement in these cases, allowing controlled tissue separation with a viscoelastic agent such as sodium hyaluronate (Healon^®^). Unlike direct membrane peeling with forceps or scissors, viscodissection enables progressive, atraumatic detachment, reducing mechanical stress on the retina and minimizing the risk of new retinal breaks. The higher viscosity and greater cohesiveness of viscoelastic in comparison to PFCL makes it a perfect material for funnel opening as it exerts a gentle yet sustained mechanical force allowing for progressive separation of the retina and creating an adequate space where the optic nerve head can be observed and PFCL can be introduced more safely. Moreover, if a small amount of viscoelastic material escapes from the funnel during dissection, it is generally less harmful than PFCL, due to its high molecular purity, non-inflammatory and biologically inert properties [12].

**Key advantages** of viscodissection in funnel retinal detachment surgery include:

1. Atraumatic funnel opening—Gentle viscoelastic injection allows for controlled separation of retinal folds, improving intraoperative visibility.
2. Reduction of iatrogenic damage—Decreases the likelihood of hemorrhages and retinal tears compared to conventional mechanical dissection.
3. Facilitation of PFCL use—Once viscodissection relaxes the retina, PFCL can be introduced more safely, minimizing the risk of subretinal migration.
4. Effective management of adhesions—Particularly useful in cases where the retina is strongly attached to the posterior capsule of the lens or the iris.

The use of viscoelastic substances is well-documented in anterior chamber surgery. However, their application in the vitreous chamber is less common, primarily for viscodissection of PVR in diabetic retinopathy [13,14,15]. Their use in retinal detachment has also been reported in the “soft shell technique”, in which viscoelastic is injected over a retinal tear to cover it and prevent the migration of subsequently injected PFCL [16]. Additionally, viscoelastic materials have been used to stabilize the retina during vitrectomy in severe cases of globe rupture [17]. The use of viscoelastic material in funnel retinal detachments remains largely unexplored, presenting a promising avenue for future research.

The use of viscoelastic material allows visualization within the funnel, making PFCL application safer. However, even after opening the funnel with viscoelastic and performing prepapillary PFCL injection, surgery remains complex. Bimanual membrane peeling or viscodissection is often required to relieve traction, enable further PFCL injection, and achieve complete retinal reattachment.

Despite its advantages, viscodissection requires precise fluid control, as excessive pressure may cause retinal tears, viscoelastic dispersion, or retention in the vitreous cavity. We have employed this technique for several years and consider it the only atraumatic approach for managing closed-funnel retinal detachments. We have not observed complications derived from this technique; however, future research should focus on optimizing it, identifying ideal viscoelastic properties, and evaluating long-term anatomical and functional outcomes.

## 5. Conclusions

In conclusion, viscodissection is a valuable addition to the vitreoretinal surgeon’s armamentarium, offering a less traumatic and more controlled approach for managing complex PVR cases with funnel detachments. Further clinical validation is needed to establish its role in standard vitreoretinal surgical protocols.

## Data Availability

The data generated for this manuscript are available from the corresponding author upon reasonable request.

## Supplementary Materials

The following supporting information can be downloaded at: https://www.mdpi.com/article/10.3390/jcm14134394/s1, Video S1: Case 3 Reduced2; Video S2: Case 5 Reduced2.
